# The transformational impact of site-specific DNA modifiers on biomedicine and agriculture

**DOI:** 10.21451/1984-3143-AR2018-0065

**Published:** 2018-08-16

**Authors:** Kathryn Polkoff, Jorge A. Piedrahita

**Affiliations:** 1 Comparative Medicine Institute, College of Veterinary Medicine, North Carolina State University, Raleigh, NC, 27606, USA.; 2 Department of Molecular Biomedical Sciences, College of Veterinary Medicine, North Carolina State University, Raleigh, NC, 27606, USA.

**Keywords:** domestic animals, gene editing

## Abstract

The development of genetically modified livestock has been dependent on incremental technological advances such as embryo transfer, homologous recombination, and somatic cell nuclear transfer (SCNT). This development rate has increased exponentially with the advent of targeted gene modifiers such as zinc finger nucleases, TAL-effector nucleases (TALENs) and clustered regularly interspaced short palindromic repeats (CRISPR-Cas). CRISPR-Cas based systems in particular have broad applicability, and have low technical and economic barriers for their implementation. As a result, they are having, and will continue to have, a transformational impact in the field of gene editing in domestic animals. With these advances also comes the responsibility to properly apply this technology so it has a beneficial effect throughout all levels of society.

## Introduction

Embryo culture and embryo transfer, pronuclear injection, homologous recombination: technical advances that gradually made it possible to generate genetically modified large animals such as pigs, cattle and sheep. While each of these incremental advances have impacted the field, two have had such a large impact as to be properly defined as transformational. They are somatic cell nuclear transfer (SCNT), and site-specific gene editing via targeted gene modifiers, including zinc finger nucleases ( Le Provost *et al*., 2010 ), TAL-effector nucleases (TALENs; Christian *et al*., 2010 ) and clustered regularly interspaced short palindromic repeats (CRISPR-Cas; Jinek *et al*., 2012 ).

Our previous inability to isolate and culture embryonic stem cells (ES) from domestic species to generate transgenic animals prevented the implementation of techniques such as homologous recombination (HR) ( Gonçalves *et al*., 2014 ; Koh and Piedrahita, 2014 ). In spite of over thirty years of work in this area, no ES cell lines from domestic species have been isolated that allow the practical and efficient generation of transgenic animals ( Koh and Piedrahita, 2014 ). Thus, while techniques such as HR using ES cells to create germ-line chimeras became the norm to generate transgenic mice, these approaches could not be used in domestic species; that is, until the advent of SCNT. From the initial observation of Keith Campbell and Ian Wilmut that sheep could be cloned from a somatic cell using SCNT ( Campbell *et al*., 1996 ), multiple groups rapidly moved to genetic modification of somatic cells *in vitro* followed by SCNT. This led to the first reports of SCNT-generated transgenic sheep ( Schnieke *et al*., 1997 ), pigs ( Dai *et al*., 2002 ) and cattle ( Cibelli *et al*., 1998 ). And these initial reports included application of HR in somatic cells before transfer ( McCreath *et al*., 2000 ). As a result, there was tremendous excitement in the field and most, if not all, laboratories worldwide working in the area of genetic modification of domestic animals quickly moved to implement SCNT. While this transition was successful for many groups, gene targeting by HR remained a significant barrier. For reasons that are still not well understood, HR in somatic cells is extremely inefficient and in spite of significant efforts by many groups, only a few gene targeted animals were generated (reviewed by Prather *et al.*, 2008 ; Aigner *et al*., 2010 ; Piedrahita and Olby, 2011 .

That all changed with the development of gene editing using targeted DNA endonucleases such as Zinc Finger Nucleases (ZFN), Tal Effector Nucleases (TALENs), and CRISPR-Cas9 nucleases ( Sander and Joung, 2014 ). All three approaches make gene targeting in any cell, including somatic cells, more efficient by several orders of magnitude ( Gaj *et al*., 2013 ). Using pigs as an example, we show in Table 1 that the impact of this technology on the efficiencies of generating a transgenic pig is indeed transformational. While all three approaches (ZNFs, TALENs, and CRISP-Cas) have been used to develop gene edited domestic species, this review will concentrate on the CRISPR-Cas based systems. This is due to the lower costs, ease of use, and expanding repertoire of modified enzymes that further increase the utility of the system. We will cover applications that focus on gene editing (genetic modifiers) as well as approaches that modify gene expression by acting on the epigenome (epigenetic modifiers). While these epigenetic modifiers have not yet been fully implement in domestic animals, we feel they have tremendous potential as models for clinical applications in humans.

**Table 1:** Effect of site-specific DNA modifiers on multiple aspects of gene editing in mammals.

Before site-directed DNA modifiers *	After site-directed DNA modifiers	References
Homologous Recombination (HR)	Homologous recombination and targeted NHEJ	Smithies, 2001; Smithies *et al*., 1984 ; Smithies, 2008 ; Le Provost *et al*., 2010
		
Long homology arms	Short or no homology arms	Vazquez *et al*., 1998; Sander and Joung, 2014 ; Suzuki *et al*., 2016 ; Brown *et al*., 2016
		
Selectable markers	No selectable markers	Smithies, 2008 ; Gaj *et al*., 2013
		
Single gene	Multiple genes	Smithies, 2008 ; Piedrahita *et al*., 1992 ; Park *et al*., 2017
		
Single allele	Both alleles	Fu *et al*., 2013
		
Only cultured cells	Cultured cells and direct zygotic injection	Dai *et al*., 2002 ; Hai *et al*., 2014
		
Overall frequency of HR 1 in a million	Overall frequency of targeted gene editing 100%	Smithies, 2008
		
Only dividing cells	Dividing and non-dividing cells	Yao *et al*., 2017
		
Global but not targeted epigenetic modifications	Single and multi loci targeted epigenetic modifications	Ng and Bird, 1999 ; Zhou *et al*., 2018
		
Frequency too low for in vivo or ex vivo clinical applications	Frequency high enough that in vivo or ex vivo clinical applications can be developed	Wang et al., 2013 ; Hai *et al*., 2014

* Includes both genetic and epigenetic modifiers.

## CRISPR-Cas editors

**Figure 1:**
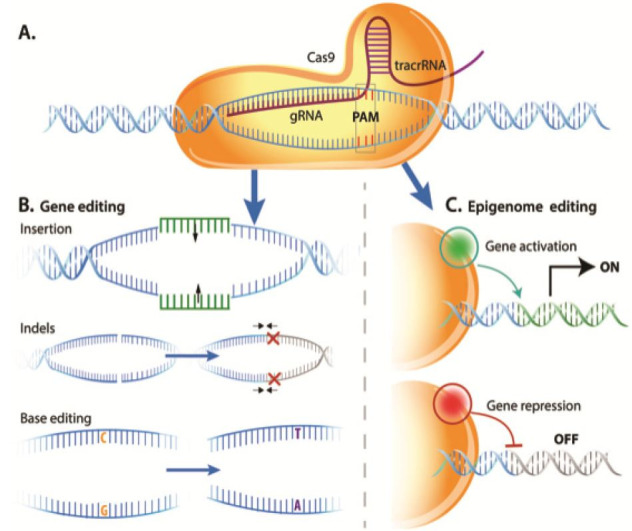
Gene editing outcomes using targeted gene modifiers. A) gRNA complexes with Cas9 protein to bind a specific 20 nucleotide sequence in the target DNA. B) Cas9 nuclease initiates cell-based repair mechanisms to create changes in DNA sequence: Homology directed repair or HITI for gene insertion, non-homologous end joining for indels, or base editing for site specific nucleotide changes. C) Catalytically inactive Cas9 protein fused with transcriptional modifiers leads to targeted gene activation or repression.

Originally derived from bacteria as a defense against bacteriophages, investigators have harnessed the ability of CRISPR-Cas to recognize a specific DNA sequence and create a double-stranded break. As depicted in Fig 1 , CRISPR-Cas has two functional components: a guide RNA (gRNA) and a C RISPR Associated protein (Cas) nuclease. The gRNA is composed of an RNA sequence that recognizes the target DNA and an RNA region known as tracrRNA or transactivating CRISPR RNA. The Cas protein complexes with the gRNA and binds the target DNA.

In the first, and most commonly used, CRISPR-Cas system derived from *S. Pyogenes* (SpCas9), the gRNA contains a 20 nucleotide sequence complementary to a DNA sequence that is directly upstream of a protospacer adjacent motif (PAM) 5’-NGG. CRISPR-Cas systems from other bacteria such as *Staphylococcus aureus* (SaCas9) and Prevotella and Francisella (Cpf1) ( Ran et al., 2015 ; Zetsche et al., 2015 ) have different gRNA sequence and PAM requirements, but all create a double-stranded break at the target site. This results in a system that can be targeted to specific regions of the genome using the gRNA followed by a double stranded DNA cleavage via the Cas9 endonuclease ( Sander and Joung, 2014 ). The cell then senses this DNA damage and activates DNA repair pathways. It is this process of DNA repair that forms the basis for gene editing using CRISPR-Cas systems. As shown in Fig. 1 , the DNA damage can be repaired by multiple mechanisms. The most frequently used, the non-homologous end joining (NHEJ) pathway, recruits cellular machinery to ligate the cleaved ends back together. However, this system is error-prone and creates random insertions and deletions (indels) at the damaged site. If the indels are located in the coding sequence of the gene, they can create a frame-shift mutation and therefore an abnormal or absent protein. Thus, NHEJ is often used to inactivate genes. This is such a highly efficient system that it can generate loss of function of one (heterozygous mutant) or both copies of the gene (homozygous mutant; Table 1 ). Prior to targeted endonucleases, the only way to obtain homozygous mutants was through breeding heterozygotes, or by performing two rounds of genetic modifications using sequential SCNT ( Kuroiwa *et al*., 2004 ); neither of which are practical or easy to apply to domestic animals. This alone is transformational as, by avoiding the need for breeding, CRISPR-Cas induced NHEJ drastically reduces the time required to generate an animal or cell line devoid of a specific protein.

However, in some cases the goal is not to knock out a gene but to instead knock in or replace genes. This process, for instance, can be used for targeted insertion of a gene such as a fluorescent tag for cell or protein tracking, insertion of human genes, or addition of favorable agricultural traits. For targeted homology directed repair, the double stranded break in the target region requires a donor DNA construct containing regions of homology to either side of the cut site. The cell will then repair the double stranded break by two competing mechanisms, the NHEJ described above, or by homology-directed repair (HDR) resulting in incorporation of the donor DNA into the target region. This process is analogous to conventional HR with the main difference being that in conventional HR there is no induced double stranded break, only the donor DNA. Both conventional HR and HDR require cell division as DNA replication is an integral component of the homologous recombination process. As described in Table 1 , without the DNA break, HDR occurs at frequencies of 0.000001% (1 in 10E56) or lower. In contrast, with a targeted DNA break, HDR occurs at frequencies ranging from 10% to as high as 50%.

But there are differences in the composition of the donor DNA as well. Conventional HR requires that the donor DNA contains several kb of homology to the target gene, as well as positive and negative selectable markers to enrich for those are cells that had been modified ( Vazquez *et al*., 1998 ). As a result, the donor DNA plasmids are difficult and expensive to develop, some requiring several months to complete. In contrast, donor DNA used for HDR requires regions of homology ranging from a total of 1 kb to less than 100 bp, does not require selectable markers, and can be rapidly and inexpensively generated. This allows the use of two types of donor DNA, small oligo that can be used to modify small regions of the DNA and larger DNA donors that can be used to replace or insert (knockin) a gene or gene fragment into the desired target region ( Sander and Joung, 2014 ).

Recently, a new mechanism for gene insertion has been described for homology independent targeted integration (HITI; Brown *et al*., 2016 ; Suzuki *et al*., 2016 ). By a process little understood at present, the double stranded break created by the CRISPR-Cas9 is repaired by an NHEJ-driven mechanism, does not require DNA replication, and results in the insertion of a donor DNA in the absence of any homology to the target region. It does, however, require that the donor DNA plasmids are also cleaved by a CRISPR-Cas9. What is more surprising is that the frequency of targeted insertions is higher using HITI that using HDR ( Suzuki *et al*., 2016 ). Since then, similar approaches using micro-homology arms (<50 bp) or homology arms of less than 1 kb of total homology have been described that also work in non-dividing cells ( Yao *et al*., 2017 ), referred to as Micro-homology Mediated End Joining (MMEJ) and Homology-Mediated End Joining (HMEJ), respectively. Interestingly, the efficiency of the different integration methods differs drastically depending on cell type. In mouse ES cells, for instance, HDR and HMEJ occur at approximately the same rate, while in mouse embryos, HMEJ is 5-10 fold more effective than HDR ( Yao *et al*., 2017 ). In summary, multiple approaches that have been or are being developed allow the modification or inactivation of essentially any gene in any cell type at high efficiency.

## Base editors

While HDR and NHEJ are effective for knock-outs and knock-ins, the advent of base editors introduces a new paradigm for therapeutic gene editing. Base editors are CRISPR-based enzymes that can catalyze the conversion of specific bases within a specified target window without a double-stranded break. The first base editors consist of a Cas9 nickase fused with a cytidine deaminase (APOBEC-1 or AID), which is directed by a gRNA to elicit a targeted C*G to T*A conversion ( Komor *et al*., 2016 ; Shimatani *et al*., 2017 ). The mechanism involves deamination of a cytidine, thereby converting it to a uridine, which pairs with an adenine upon cellular repair. Further iterations of base editors also allow for improved specificity of the target window to eliminate unintended conversion of cytosines neighboring the target base pair ( Kim *et al*., 2017 ). More recent advances also allow the conversion of T*A pairs to C*G pairs by replacing the cytidine deaminase with an adenosine deaminase ( Gaudelli *et al*., 2017 ). In addition, base editors have been fused to other targeted endonucleases such as Cpf1 ( Li *et al*., 2018 ), allowing for targeting of sites with various PAM sequences.

The use of base editors has several advantages: there is no longer a need to simultaneously deliver a repair template with the endonuclease, the lack of double-stranded breaks diminishes the chance of unwanted indels, and the specific activity window reduces the number of potential off-target sites (discussed below). However, base editors are still restricted in that they only can catalyze conversions between C*G and T*A base pairs, and only can target a small window which must be approximately 15 base pairs upstream of the PAM sequence. Future base editors must be more flexible to allow for editing of clinically relevant sites that are currently out of reach due to lack of appropriate PAM location or that require different base conversions. Furthermore, microinjection of a base editor into mouse embryos showed that its nickase activity can still introduce indels at a relatively high frequency ( Kim *et al*., 2017 ).

## Approaches to generating gene edited offspring

Unlike conventional HR, where the efficiencies are so low that *in vivo* applications in embryos or somatic tissue are impractical, the increases in gene editing frequencies associated with systems such as CRIPS-Cas make *in vivo* gene editing possible. While gene editing was initially carried out in cells in culture, *in vivo* applications quickly developed. Initial reports showed that direct injection of CRISPR-Cas9 into the cytoplasm of one cell mouse embryos resulted in 50/56 (90%) of the offspring being modified via NHEJ ( Wang *et al*., 2013 ). What was more surprising was the large number of offspring that had biallelic modifications 45/56 (80%). These initial reports were soon confirmed in other species including domestic animals such as pigs ( Hai *et al*., 2014 ), sheep ( Crispo *et al*., 2015 ), goats ( Wang *et al*., 2015 ) and cattle ( Bevacqua *et al*., 2016 ). It was also applied to multiple loci at the same time resulting in the generation of multi transgenic offspring ( Park *et al*., 2017 ).

Initial reports of HDR by direct cytoplasmic injection were not as successful as NHEJ, suggesting that homologous recombination is not efficient in embryos, with frequencies ranging from 8 to 34% in mice ( Yang *et al*., 2013 ). Approaches to enhance HDR were tested ( Maruyama *et al*., 2015 ) with some success but it was not until NHEJ-dependent approaches were used that targeted insertion into zygotes became practical. The MHEJ system already described is highly efficient when used directly on embryos with over 25% of mouse embryos carrying the correct insertion and over 50% of non-human primate embryos generated by ICSI, followed by HMEJ, carrying the correct modification ( Yao *et al*., 2017 ). In domestic species, HDR directly in embryos has been reported for pigs ( Park *et al*., 2017 ) and goats ( Niu *et al*., 2018 ) with efficiencies ranging from 15 to 50%. Of concern, however, is that in addition to the HDR-mediated insertion into one allele, the remaining allele was mutated via NHEJ. Thus, a large number of offspring need to be generated to create one carrying the desired insertion but without a mutation in the other allele.

This leads to the question: which system is better, gene editing directly in zygotes or gene editing in cultured cells followed by SCNT? There is no simple answer. It depends on the question being addressed, the technical capabilities available, and the regulatory environment in which one operates. The benefits of gene editing combined with that SCNT is that the donor cells can be extensively analyzed before SCNT so the genetics of the offspring are known. It also allows for complex gene edits and for sequential gene editing via multiple rounds of SCNT. This allows generation of multi-transgenic animals in a relatively short time, something that is crucial in species with longer generational intervals (cattle, goats, and pigs for instance). The drawbacks are that SCNT is technically complex, requires expensive specialized equipment, and can be unreliable. Zygotic injection, in contrast, is technically simple, can be carried out with less expensive equipment, and can be applied, in theory, to any mammalian species where zygotes are available; even those where SCNT is either impractical or has not been developed. The drawback is that the process is completely random so many of the offspring generated will have to be euthanized as they will not carry the desired gene edit. In addition, it cannot be used to generate sequential gene edits without breeding to produce new zygotes and that in species such as cattle can take years rather than months. However, if all that is needed is inactivation of 1-2 genes or modification of one loci, zygotic injection will produce the desired outcomes in a shorter period of time, even if some of the offspring do not carry the desired mutation and will need to be discarded. In an ideal system, having both SCNT and zygotic injection will give the greatest flexibility and provide the capabilities to tackle essentially any gene edit desired, regardless of complexity.

## Epigenetic modifiers

Although gene targeting for knock-outs, knock-ins, and editing is very promising, there is also a need to address diseases that are a result of aberrant cellular regulation. In the past decades, modulation of gene expression has depended heavily on RNA interference, which focuses mostly on gene repression ( Gemberling and Gersbach, 2018 ). The development of CRISPR-based gene regulators provides a powerful new strategy for targeted gene therapy. These epigenomic editors are composed of a catalytically inactive CRISPR protein, dead-Cas9 (dCas9), fused with an effector domain for transcriptional activation or suppression. These complexes are then paired with a gRNA and targeted to a specific site in the genome. With control of transcriptional activity, these editors can be used to suppress harmful genes, upregulate those that are deficient or silenced, or completely reprogram cell fate.

A series of dCas9 transcriptional activators have been developed, the first of which were dependent on the transcriptional activator VP64 ( Gilbert *et al*., 2013 ; Perez-Pinera *et al*., 2013 ). Improved versions depend on addition of fused domains, protein scaffolds, or RNA scaffolds to recruit additional upregulating factors for improved efficiency ( Chakraborty *et al*., 2014 ; Konermann *et al*., 2015 ; Chavez *et al*., 2016 ). Other dCas9 activators rely on epigenomic modifiers such as histone acetyltransferase ( Hilton *et al*., 2015 ). As for transcriptional repressors, early versions relied upon dCas9 binding to interfere and block transcription initiation ( Qi *et al*., 2013 ). Soon after, dCas9 was used to recruit chromatin-modifying repressor complexes, such as the Kruppel-associated box (KRAB) domain, to effectively silence target gene expression ( Gilbert *et al*., 2013 ). Moreover, because specific effects of epigenetic elements on gene regulation are not well understood, targeted epigenetic modifications by DNA methyltransferase ( Vojta *et al*., 2016 ), or histone deacetylase ( Kwon *et al*., 2017 ) can be employed to better understand these phenomena. This type of screen for regulatory elements can also be performed in a high-throughput fashion with loss- and gain-of-function editors ( Klann *et al*., 2017 ).

Like their active-nuclease counterparts, dCas9 epigenome modifiers can also be delivered for therapeutic and fundamental purposes. Several studies have shown the ability of CRISPR activators to modify cell fate. For example, *in vitro* studies have demonstrated effective direct reprogramming of fibroblasts into neurons by targeted activation of three specific genes ( Black *et al*., 2016 ). These factors can also be delivered *in vivo* by the same approaches as the targeted nucleases, such as AAV. An impressive study by Liao *et al*. (2017) was the first to use CRISPR/Cas9 type systems to modify transcription for several purposes. They show the ability to increase muscle mass in a dystrophic mouse model by local injection into hindlimbs by upregulating utrophin, compensate for acute kidney injury by upregulating Klotho or IL-10, and completely reprogram liver cells into insulin producing cells to treat a mouse model of type 1 diabetes. This is the first of many future studies using *in vivo* transcriptional modifiers as therapeutics for disease and perhaps for production or reproductive traits in large animals.

Other uses for dCas9 delivery include reprogramming of astrocytes into neurons in transgenic mice by activation of multiple genes ( Zhou *et al*., 2018 ) or the ability to screen for potential oncogenes ( Chow and Chen, 2018 ). Because they are so new, the *in vivo* delivery of targeted transcriptional regulators has thus far been limited to small animals, but as the therapies are translated to humans, we expect large animal models such as pigs to be important for scale-up and evaluation of physiological effects. Pigs have already been established as a model for epigenetic programming. For example, an Oct4-Enhanced GFP pig provides a valuable tool for the evaluation of reprogramming efficiency and pluripotency ( Nowak-Imialek *et al*., 2010 ). Even in a pre-targeting era, pigs have been useful for the study of epigenetic control of gene expression, silencing, or tissue specific control of transgenes ( Archer *et al*., 2003 ; Kues *et al*., 2006 ).

## Hurdles and challenges: off-target effects

One major limitation for the use of CRISPR is the potential for off-target effects. While each gRNA has been synthesized to target a specific genomic sequence, there is the possibility for binding and cleavage at closely related sequences elsewhere in the genome, resulting in unwanted indels. The presence of off-target effects from CRISPR-Cas was shown in human cells early on ( Fu *et al*., 2013 ), and hence there has been a push to develop methods for detection and prevention of off-target effects.

Initial efforts for safe and effective CRISPRs led to in silico design tools for gRNAs that score the probability of on- and off-target events ( Hsu *et al*., 2013 ; Heigwer *et al*., 2014 ). While these are a good starting point and are free to use, in silico design tools are only moderately accurate for prediction of true off-target effects ( Tsai *et al*., 2015 ). To better understand the frequency and location of off-target sites, a handful of techniques have been established. GUIDE-seq ( Tsai *et al*., 2015 ), CIRCLE-seq ( Tsai *et al*., 2017 ), Digenome-seq ( Kim *et al*., 2015 ), and HTGTS ( Frock *et al*., 2015 ) are all examples of unbiased, sensitive tools that capture the double-stranded breaks created with *in vitro* or in situ following delivery of Cas9 and analyze based on sequence reads. However, these techniques are expensive and frequently require a full reference genome. It is of note that off-target sites detected by GUIDE-seq showed only modest overlap with in silico predictors especially because many actual off-target sites were excluded from consideration by the programs ( Tsai *et al*., 2015 ). As studies continue to elucidate the precise rules for CRISPR off-target binding ( Boyle *et al*., 2017 ), there is a need for a more accurate in silico

predictor tool. Likewise, off-target effects are highly characterized *in vitro* , but more work must be done to evaluate frequency of off-target events *in vivo* .

To combat these adverse effects, several strategies have been employed. Initial studies demonstrate that a shortened gRNA can increase specificity by eliminating ability to form bulges when binding ( Fu *et al*., 2014 ). Further studies showed that the use of Cas9 nickases or paired nickases, which create only a single-stranded break, have fewer off-target effects ( Shen *et al*., 2014 ; Frock *et al*., 2015 ). Additionally, modifications to the Cas9 protein for a high fidelity nuclease increase the specificity of the binding domain and decrease off-target effects and frequency ( Kleinstiver *et al*., 2016 ). Finally, other CRISPR nucleases with less common PAM sequences or that are less tolerant of mismatches, such as Cpf1, have fewer off-target sites compared with Cas9 ( Kim *et al*., 2016 ). The availability of new enzymes with higher fidelity and higher specificity combined with better in silico methods to design gRNA that will have single target specificity are likely to eventually lead to systems with undetectable off-target effects. For the present, however, it is important that off-target effects are taken into account when generating gene edited offspring, whether by SCNT or by zygotic injection.

## Summary and conclusions

With the rapid adaptation of CRISPR-Cas and related gene editing technologies, the rate of applications to agriculture and biomedicine is growing exponentially. Previous methods of genetic modification of animals relied heavily on random insertion methods (pronuclear injection), use of genetically modified somatic cells followed by SCNT, or the use of viruses for transgene insertion; all methods with significant drawbacks. CRISPR-Cas and related systems not only do not suffer from these drawbacks but their implementation is both technically simpler and less costly. All these factors combined, and the high degree of plasticity of the procedure so it can be used to modify DNA as well as modify transcription, is transforming the field of gene editing of domestic animals.

However, as we continue to apply gene editors, whether it be for therapeutic delivery in medicine or disease resistance and growth traits in agriculture, we must be responsible and aware of our actions. The power of this technology is immense, and any misuse of it will decrease acceptance from the public who needs it the most. Nevertheless, proper use of these tools brings us the opportunity to cure disease, improve agricultural production to feed the growing population, and create a healthy future.
